# Clinical application of metagenomic next-generation sequencing in etiologic diagnosis of severe pneumonia in adults

**DOI:** 10.3389/fcimb.2025.1561468

**Published:** 2025-04-28

**Authors:** Zhen-Chuan Xing, Hua-Zheng Guo, Peng Zhen, Ting Ao, Ming Hu

**Affiliations:** ^1^ Department of Pulmonary and Critical Care Medicine, Beijing Luhe Hospital, Capital Medical University, Beijing, China; ^2^ Department of Infectious Disease, Beijing Luhe Hospital, Capital Medical University, Beijing, China

**Keywords:** severe pneumonia, adults, metagenomics, next-generation sequencing, pathogen detection

## Abstract

**Objective:**

To analyze the clinical characteristics and risk factors for death of severe pneumonia (SP) in adults and explore the application value of metagenomic next-generation sequencing in the detection of pathogens.

**Methods:**

A total of 132 adult patients with SP admitted from May 2021 to October 2023 were selected. Data on gender, age, smoking, underlying diseases, laboratory tests and prognosis were collected. BALF samples were sent for mNGS, smear-stained microscopy and culture. Meanwhile, conventional methods were used for pathogen detection of blood, urine and throat swab specimens. The detection efficiencies of different methods were compared and the associated pathogen profiles were analyzed.

**Results:**

Among the 132 patients, there were 92 males and 40 females, with a total of 52 deaths. Age≥65 years, heart failure, renal insufficiency, positive of COVID-19, use of vasoactive drugs, use of mechanical ventilation and use of CRRT were statistically different between the survivors and non-survivors. Heart failure (OR=4.751) and use of mechanical ventilation (OR=11.914) were risk factors of SP mortality. The bacteria detected were mainly Klebsiella pneumoniae, Acinetobacter baumannii and Pseudomonas aeruginosa. The fungi detected were mainly Candida and Aspergillus. The viruses detected were mainly COVID-19 and influenza virus. The positive rate of mNGS was higher than conventional methods in both bacteria, fungus and virus (82.58% vs 63.64%, 50.76% vs 37.88% and 67.42% vs 37.88%, respectively) (*P*<0.05). The sensitivity and accuracy of mNGS in bacterial detection were significantly higher than traditional methods (*P*<0.05). Compared to culture, mNGS detected more Staphylococcus aureus, Streptococcus pneumoniae, Haemophilus influenzae and Escherichia coli, and had a significant advantage in the detection of Mycobacterium tuberculosis complex, Nontuberculous mycobacterial, Legionella pneumophila, Chlamydia psittaci, Pneumocystis jirovecii and Aspergillus. Moreover, mNGS can better indicate mixed infections of bacteria, viruses, or fungi.

**Conclusion:**

Elderly people with chronic diseases were the main group of severe pneumonia in adults. The pathogenic microorganisms that caused SP are complex, and mixed infection is common. mNGS enhanced the effectiveness of pathogen detection, makes up for the shortcomings of conventional methods, especially in identifying unexpected pathogens, and provides a new means for early targeted anti-infection treatment.

## 1 Introduction

Severe pneumonia (SP) has been one of the major concerns of the medical community. Literature reports that the 30-day mortality rate of SP is 23% to 47% (Mortensen et al., 2004; Restrepo et al., 2008; Restrepo et al., 2010; Hraiech et al., 2013), and it is the main cause of death in patients in intensive care units. The report from the World Health Organization shows that among the 10 leading causes of death globally in 2021, COVID-19 and lower respiratory infections ranked second and fifth respectively (World Health Organization, 2024). The clinical manifestations of SP are diverse, and the pathogens vary among different groups. Pathogen identification and initiation of targeted anti-infective therapy as soon as possible are essential to reduce mortality, improve prognosis, and reduce medical costs. Traditional diagnostic techniques in the microbiology laboratory include microscopic examination of a smear stain, growth and isolation of microorganisms in culture, detection of pathogen-specific antibodies (serology) or antigens. These methods have limitations, such as cumbersome to operate, difficulty in culturing specific pathogenic bacteria, longer time required, and lower sensitivity, which cannot meet the needs of clinical work.

Metagenomic next-generation sequencing (mNGS) technology is a microbial identification technology that has emerged in recent years. It approaches characterize all DNA or RNA present in a sample, enabling analysis of the entire microbiome in patient samples and rapid diagnosis of clinically pathogenic microorganisms (Rossen et al., 2018; Chiu and Miller, 2019). Compared with other methods, mNGS has the advantages of wide pathogen coverage, short time-consumption and high sensitivity. In this study, 132 adult patients with SP were analyzed in our hospital. The mNGS technology was compared with conventional diagnostic methods to analyze pathogen spectrum and evaluate the value of mNGS in SP.

## 2 Materials and methods

### 2.1 Study subjects

A total of 132 adult patients with SP, who were admitted to Department of Infectious Disease, Beijing Luhe Hospital, Capital Medical University from May 2021 to October 2023, were enrolled in this study. Inclusion criteria: (i) The diagnosis of SP meets the criteria established by the American IDSA/ATS (Lim et al., 2009); (ii) Conventional immunological methods were used to conduct etiological tests on blood, urine, and throat swab specimens; (iii) BALF was sent for mNGS, smear staining microscopy and cultures at the same time. Exclusion criteria: (i) Patients under 18 years old; (ii) Unqualified specimens and incomplete clinical data. Clinical data of each patient were obtained from the electronic medical record system. The study was approved by the hospital ethics committee (2021-LHKY-065-01).

### 2.2 Clinical data

The general clinical information extracted included: age, gender, smoking history, past medical history (heart failure, stroke, diabetes, malignant cancer, chronic lung disease, renal insufficiency); whether there was invasive mechanical ventilation, continuous renal replacement therapy (CRRT)), and vasoactive agent use; hospital length of stay and outcomes.

The conventional microorganism detection technologies applied: (i) antigen testing (include Influenza A antigen, Influenza B antigen, streptococcus pneumoniae urinary antigen tests, (1, 3)-β-D-glucan and galactomannan (*G/GM*) tests); (ii) serological antibody detection (include Influenza A antibody, Influenza B antibody, Mycoplasma pneumoniae antibody, Chlamydia pneumoniae antibody, Respiratory syncytial virus antibody, Legionella pneumophila antibody, Parainfluenza virus antibody and Adenovirus antibody); (iii) acid-fast staining of bronchoalveolar lavage fluid specimens; (iv); culture of bronchoalveolar lavage fluid specimens; (v) COVID-19 nucleic acid testing of nasopharyngeal swabs specimens.

### 2.3 mNGS and bioinformatics analysis

For the BALF samples, cell-free DNA was extracted using the QIAamp DNA Micro Kit (QIAGEN, Hilden, Germany) (DNA process), or RNA was extracted using the QIAamp Viral RNA Mini Kit (QIAGEN, Hilden, Germany) and then reverse transcribed it into DNA (RNA process), and then libraries were constructed using the QIAseq Ultralow Input Library Kit for Illumina (QIAGEN, Hilden, Germany), and quality control of the libraries was carried out using Qubit^®^ (Thermo Fisher Scientific, Massachusetts, USA) and Agilent 2100 Bioanalyzer (Agilent Technologies, Palo Alto, California, USA). Qualified libraries were sequenced on the NextSeq 550 System (Illumina, San Diego, California, USA).

For the bioinformatics analyses, short, low-quality, low-complexity and adapter-contaminated reads were filtered out from the raw data. Data retained after filtering were mapped to a human reference database (hg38) to remove human host DNA sequences. The remaining data were then aligned against a microbial genome database using the Burrows-Wheeler Aligner (BWA) tool to determine the microbial composition of the sample.

### 2.4 Statistical analysis

All statistical analyses were performed by using SPSS (version 25.0). Continuous variables with normal distribution were represented by mean ± standard deviation (mean ± SD), and inter-group comparisons were performed using independent sample t-test. Continuous variables with non-normal distribution were expressed as median [interquartile range] (median [IQR]), and inter-group comparisons were performed using Mann-Whitney U test. Categorical variables were expressed as frequency and percentage [N(%)], and inter-group comparisons were performed using Pearson chi-square test (expected frequency≥5) or Fisher’s exact test (expected frequency<5). Multivariate analysis was performed by binary logistic regression. The diagnostic performance of mNGS and conventional methods for the final clinical diagnosis was evaluated by using sensitivity, specificity, positive predictive value (PPV), negative predictive value (NPV) and accuracy. Two-tailed *P* values < 0.05 were considered statistically significant.

## 3 Results

### 3.1 Patient baseline characteristics

A total of 132 patients with SP were enrolled in our study, including 92 males and 40 females. The age of patients ranged from 23 years old to 97 years old, with a median age of (70.07 ± 13.66) years. The length of hospital stay ranged from 1 day to 93 days, with an average of 20 (10, 26) days. Among the 132 patients, 66 cases received invasive mechanical ventilation, of which 49 patients had invasive ventilation time≥96h, 12 patients received renal replacement therapy, 61 patients used vasoactive drugs (**Table 1**).

**Table 1:** Characteristics of 132 patients with severe pneumonia.

Characteristics	Value n (%)
Age [years, ( $\bar{x}$ ± s)]	70.07 ± 13.66
>65 years	92 (69.70%)
≤65 years	40 (30.30%)
Sex	
Male	92 (69.70%)
Female	40 (30.30%)
History of smoking	59 (44.70%)
Medical history	
Heart failure	63 (47.73%)
Stroke	46 (34.85%)
Diabetes	42 (31.82%)
Chronic lung disease	30 (21.13%)
Malignant cancer	20 (15.15%)
Renal insufficiency	14 (10.61%)
ICU treatment	
Use of vasoactive drugs	61 (46.21%)
Use of invasive mechanical ventilation	66 (50.00%)
CRRT	12 (9.10%)
Hospital LOS [days, median (IQR)]	20 (10, 26)
Outcomes	
Improved	80 (60.60%)
Died	52 (39.40%)

ICU, intensive care unit; CRRT, continuous renal replacement therapy; LOS, length of stay; IQR, interquartile range.

### 3.2 Comparison between the survival group and the death group

Of the 132 patients, 80 survived and 52 died. The univariate analysis showed that age≥65 years, heart failure, renal insufficiency, positive of COVID-19, use of vasoactive drugs, use of mechanical ventilation and use of CRRT were statistically different between the survivors and non-survivors (*P*<0.05) (**Table 2**). Binarylogistic regression analysis suggested that heart failure (*OR*=4.751) and use of mechanical ventilation (*OR*=11.914) were independent risk factors for death in patients with SP (*P*<0.05) (**Table 3**).

**Table 2:** Analysis of factors affecting the prognosis of SP patients. .

	Survivors (n=80)	Non-survivors (n=52)	χ^2^	*P*
Age>65 years	50 (62.50%)	42 (80.77%)	4.980	0.026
Male	54 (67.50%)	38 (73.08%)	0.464	0.496
History of smoking	35 (43.75%)	23 (44.23%)	0.003	0.957
Heart failure	19 (23.75%)	43 (82.69%)	43.957	0.000
Stroke	24 (30.00%)	21 (40.38%)	1.513	0.219
Diabetes	23 (28.75%)	19 (36.54%)	0.881	0.348
Chronic lung disease	20 (25.00%)	10 (19.23%)	0.597	0.440
Malignant cancer	12 (15.00%)	8 (15.38%)	0.004	0.592
Renal insufficiency	2 (2.50%)	12 (20.69%)	14.074	0.000
Positive of COVID-19	14 (17.50%)	23 (44.23%)	11.163	0.001
Use of vasoactive drugs	31 (38.75%)	30 (57.69%)	4.549	0.033
Use of mechanical ventilation	21 (26.25%)	45 (86.54%)	45.819	0.000
CRRT	2 (2.50%)	10 (19.23%)	10.674	0.001

**Table 3:** Risk factors for mortality.

	*OR*	95%*CI*	*P*
Age>65 years	1.194	(0.374 – 3.818)	0.765
Heart failure	4.751	(1.630 – 13.850)	0.004
Renal insufficiency	7.105	(0.889 – 56.813)	0.065
Positive of COVID-19	3.378	(0.933 – 12.226)	0.064
Use of vasoactive drugs	2.605	(0.935 – 7.256)	0.067
Use of mechanical ventilation	11.914	(3.662 – 38.766)	0.000
CRRT	1.881	(0.176 – 20.044)	0.601

### 3.3 Different types of pathogens detected by mNGS and traditional methods

The mNGS portend significantly higher etiological detection rate compared with traditional detection methods: bacteria (82.58% vs 63.64%), fungi (50.76% vs 37.88%), viruses (67.42% vs 37.88%), and the extent of variation was statistically significant (*P*<0.05) (**Table 4**).

**Table 4:** Comparison of conventional methods and mNGS in different types of pathogens.

	mNGS(n=132)	Conventional methods(n=132)	χ^2^	*P*
Bacteria	109(82.58%)	84(63.64%)	12.041	0.001
Fungi	67(50.76%)	50(37.88%)	4.436	0.035
Viruses	89(67.42%)	50(37.88%)	23.110	0.000

**Figure 1:**
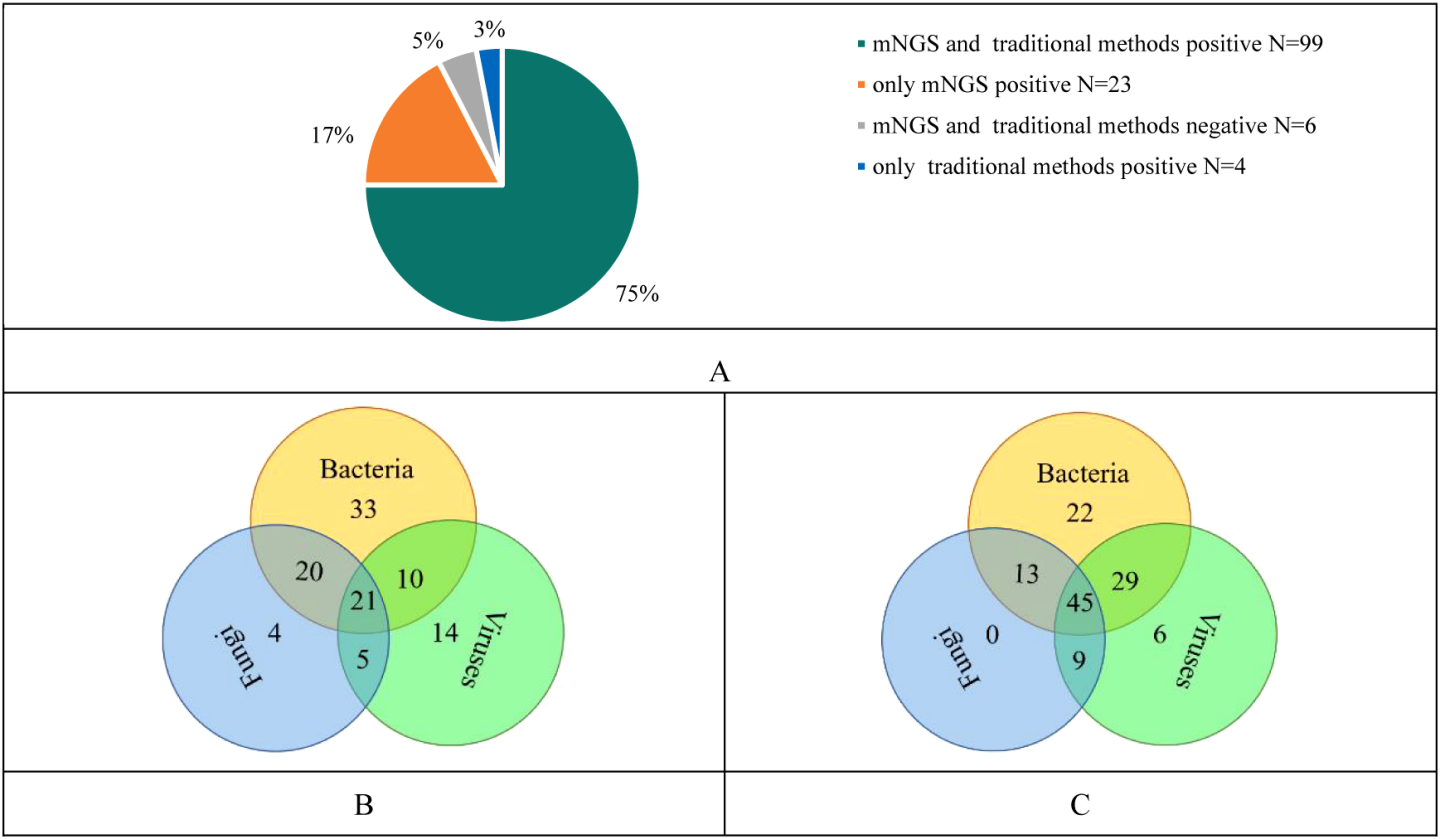
**(A)** Consistency analysis of mNGS and traditional methods results; **(B)** Pathogenic microorganisms detected by traditional methods; **(C)** Pathogenic microorganisms detected by mNGS.

The results of our study revealed 99 cases were positive in both mNGS and conventional methods, 23 cases were positive in mNGS only, 4 cases were positive in conventional methods only and 6 cases were negative in both mNGS and conventional methods (**Figure 1A**).

The conventional method showed that only bacteria were detected in 33 cases, only fungi in 4 cases and only viruses in 14 cases. Twenty-one cases showed infection with three pathogens including bacteria, fungi and viruses (**Figure 1B**). While, mNGS showed that only bacteria were detected in 22 cases, only fungi in 0 case, and only viruses in 6 cases. Forty-fifth cases showed infections with three pathogens (**Figure 1C**). Compared with traditional methods, mNGS was more likely to indicate mixed infections in cases.

#### 3.3.1 Detection of bacteria

**Figure 2:**
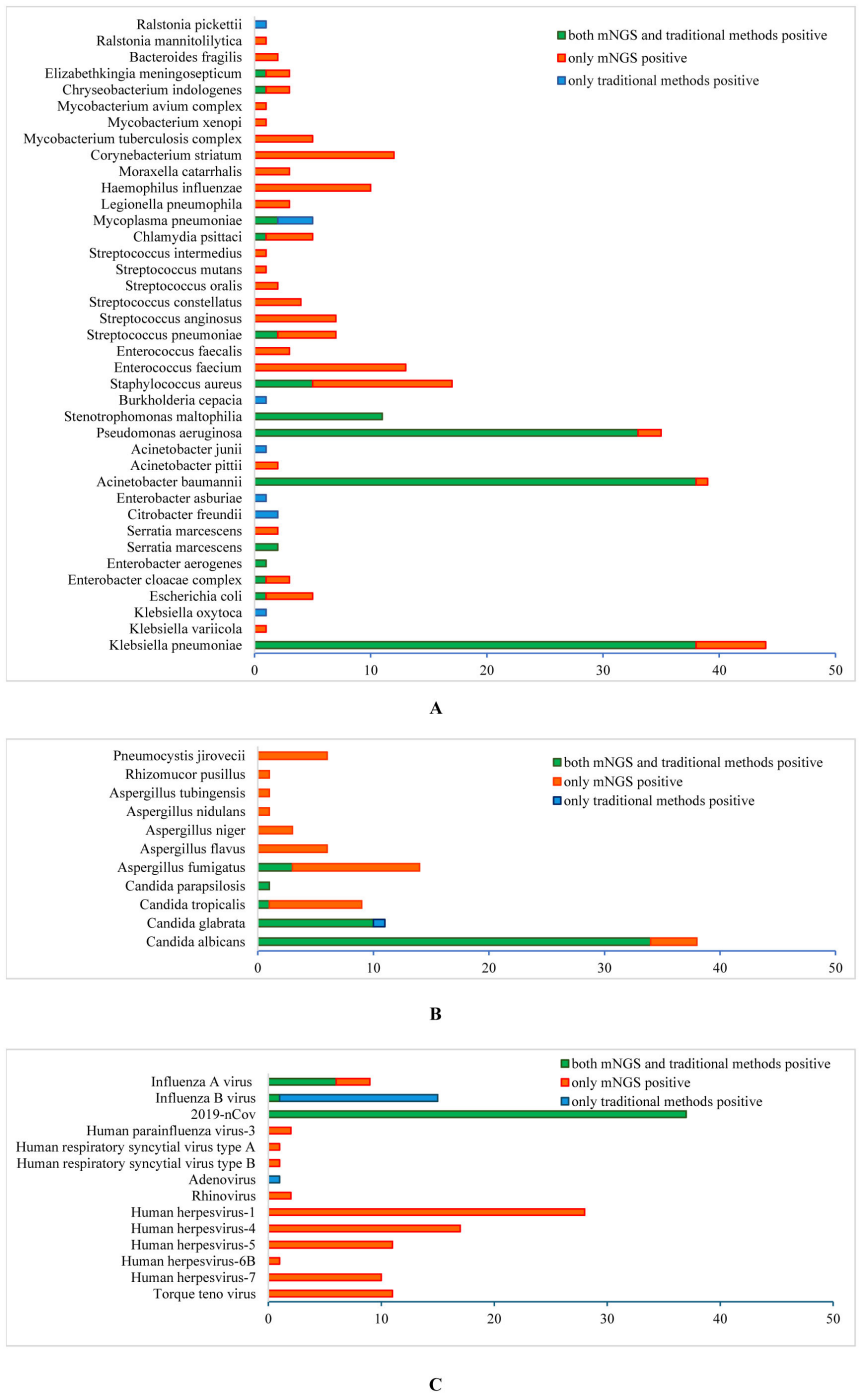
Different pathogenic organisms (in numbers) in mNGS and conventional methods. **(A)** Bacteria detected by mNGS and conventional methods; **(B)** Fungi detected by mNGS and conventional methods; **(C)** Viruses detected by mNGS and conventional methods.

Both traditional methods and mNGS showed that the most common pathogens were Klebsiella pneumoniae(38 vs 44), Acinetobacter baumannii(38 vs 39), Pseudomonas aeruginosa (33 vs 35), Stenotrophomonas maltophilia(11 vs 20) and Staphylococcus aureus(5 vs 17). Other bacteria detected by conventional methods and mNGS included Streptococcus pneumoniae(2 vs 7), Mycoplasma pneumoniae(5 vs 2), Chlamydia psittaci(0 vs 5), Chlamydia pneumoniae(1 vs 0), Escherichia coli (1 vs 5), Proteus mirabilis(2 vs 2), Elizabethkingia meningosepticum(1 vs 3), Chryseobacterium indologenes(1 vs 3), Enterobacter cloacae complex(1 vs 3), and Enterobacter aerogenes(1 vs 1). Bacteria detected only by mNGS were Enterococcus (3 cases of *E. faecalis* and cases of 13 *E. faecium*), Corynebacterium striatum(12 cases), Haemophilus influenzae(10 cases), Mycobacterium(5 cases of *MTBC*, 1 case of *MAC* and 1 case of *M. xenopi*), Streptococcus(7 cases of *S. anginosus*, 4 cases of *S. constellatus*, 2 cases of *S. oralis*, 1 case of *S. mutans* and 1 case of *S. intermedius*), Legionella pneumophila(3 cases), Moraxella catarrhalis(3 cases), and Bacteroides fragilis(2 cases) (**Figure 2A**).

#### 3.3.2 Detection of fungi

Candida was the predominant fungus detected by culture and mNGS, including *C. albicans*(34 vs 38), *C. glabrata*(11 vs 10), *C. tropicalis*(1 vs 9) and *C. parapsilosis*(1 vs 1). Meanwhile, filamentous fungal were detected by culture in 3 cases, and Aspergillus fumigatus in 14, *A. flavus* in 6, *A. niger* in 3, *A. tubingensis* in 1, *A. nidulans* in 1, Rhizomucor pusillus in 1 and Pneumocystis jirovecii in 6 were detected by mNGS (**Figure 2B**). Ninety-nine cases underwent traditional *G/GM* test, which showed 3 positive in G test alone, 10 positive in *GM* test alone, and 10 positive in both *G* and *GM* tests.

#### 3.3.3 Detection of viruses

In terms of virus detection, the main viruses detected by traditional methods and mNGS were 2019-nCov (37 vs 37), influenza A virus (6 vs 9), and influenza B virus (15 vs 1). One case of adenovirus was detected by the conventional method alone, and the viruses detected by mNGS alone included 67 cases of human herpesviruses (28 cases of type 1, 17 cases of type 4, 11 cases of type 5, 1 case of type 6B and 10 cases of type 7), 11 cases of Torque teno virus, 2 cases of human parainfluenza virus-3, 2 cases of rhinovirus-A, 2 cases of human respiratory syncytial virus (1 case of type A and 1 case of type B) and 1 cases of JC polyomavirus (**Figure 2C**).

#### 3.3.4 Pathogen co-infection

**Figure 3:**
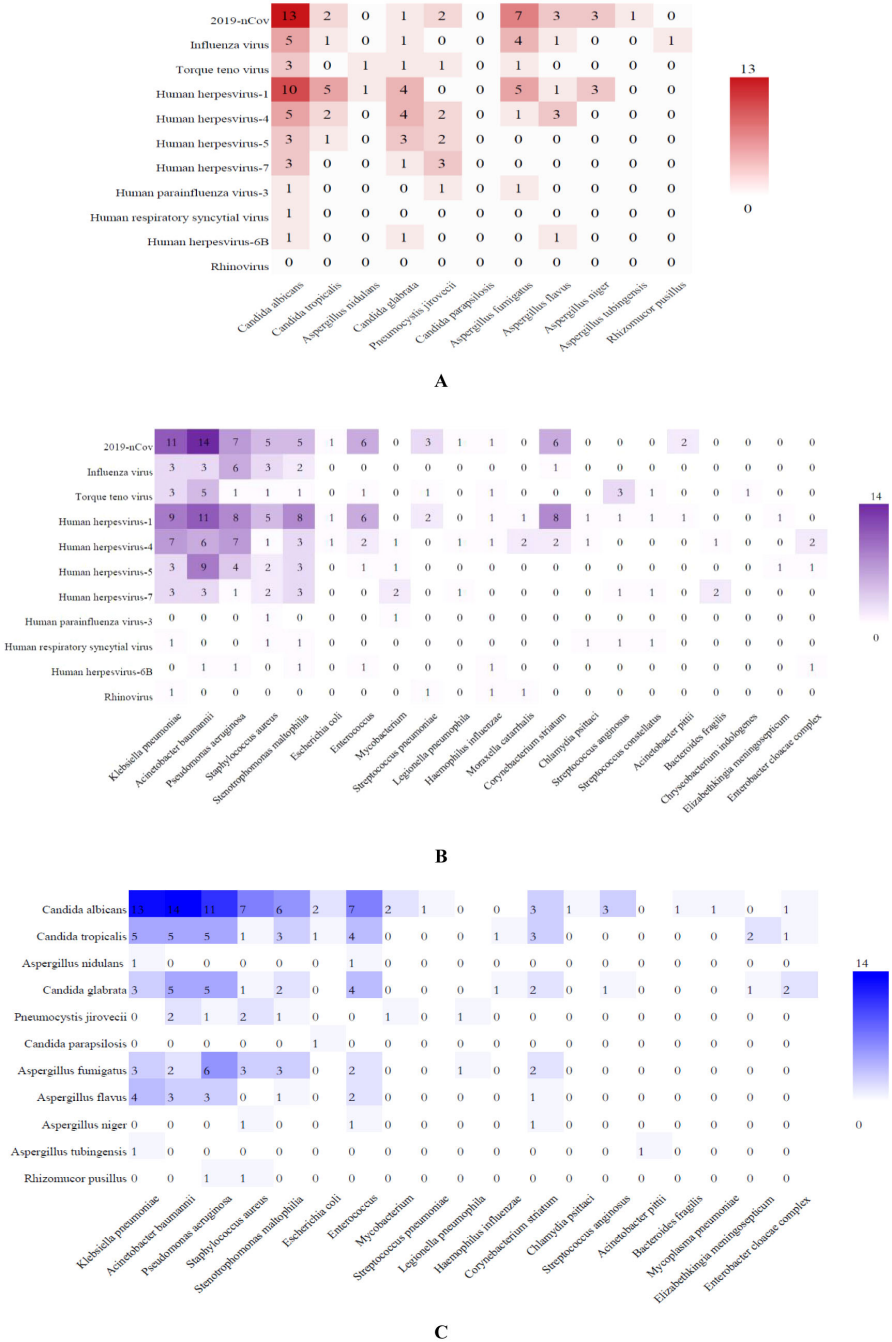
Heatmap of the co-infection of respiratory pathogens. The grid color represents the co-infection numbers of pathogens among patients with SP. Darker color of the grid indicates higher co-infection numbers between the pairs of pathogens. **(A)** Viral-fungal co-infections; **(B)** Viral-bacterial co-infections; **(C)** Fungal–bacterial co-infections.

Compared with traditional methods, mNGS detected more mixed infections. Viral-fungal co-infections detected by mNGS were predominantly 2019-nCov combined with *C. albicans* (13 cases), 2019-nCov combined with Aspergillus (13 cases), Influenza virus combined with *C. albicans* (5 cases), Influenza virus combined with *A. fumigatus* (4 cases), Human herpesviruses (HHVs) combined with Candida (42 cases), HHVs combined with Aspergillus (13 cases) and HHVs combined with Penumocysts jirovecii (7 cases) (**Figure 3A**). Viral-bacterial co-infections were mainly 2019-nCov with *A. baumanninii* (14 cases), *K. pneumoniae* (11 cases), *P. aeruginosa* (7 cases), Enterococcus (6 cases), *C. striatum* (6 cases), *S. aureus* (5 cases) and *S. maltophilia* (5 cases). Human herpesvirus combined with *A. baumanninii* (29 cases), *K. pneumoniae* (22 cases), *P. aeruginosa* (20 cases), *S. maltophilia* (17 cases), *S. aureus* (10 cases), *C. striatum* (10 cases), Enterococcus (9 cases). Influenza virus combined with *P. aeruginosa* (6 cases), *K. pneumoniae* (3 cases), *A. baumanninii* (3 cases) and *S. aureus* (3 cases) (**Figure 3B**). Fungal–bacterial co-infections were mostly Candida with *A. baumanninii* (24 cases), *K. pneumoniae* (21 cases), *P. aeruginosa* (21 cases), Enterococcus (15 cases), *S. aureus* (11 cases) and *S. maltophilia* (11 cases); and Aspergillus with *K. pneumoniae* (9 cases), *P. aeruginosa* (9 cases), Enterococcus (6 cases) and *A. baumanninii* (5 cases) (**Figure 3C**).

### 3.4 Clinical diagnostic performance of mNGS and conventional methods

**Figure 4:**
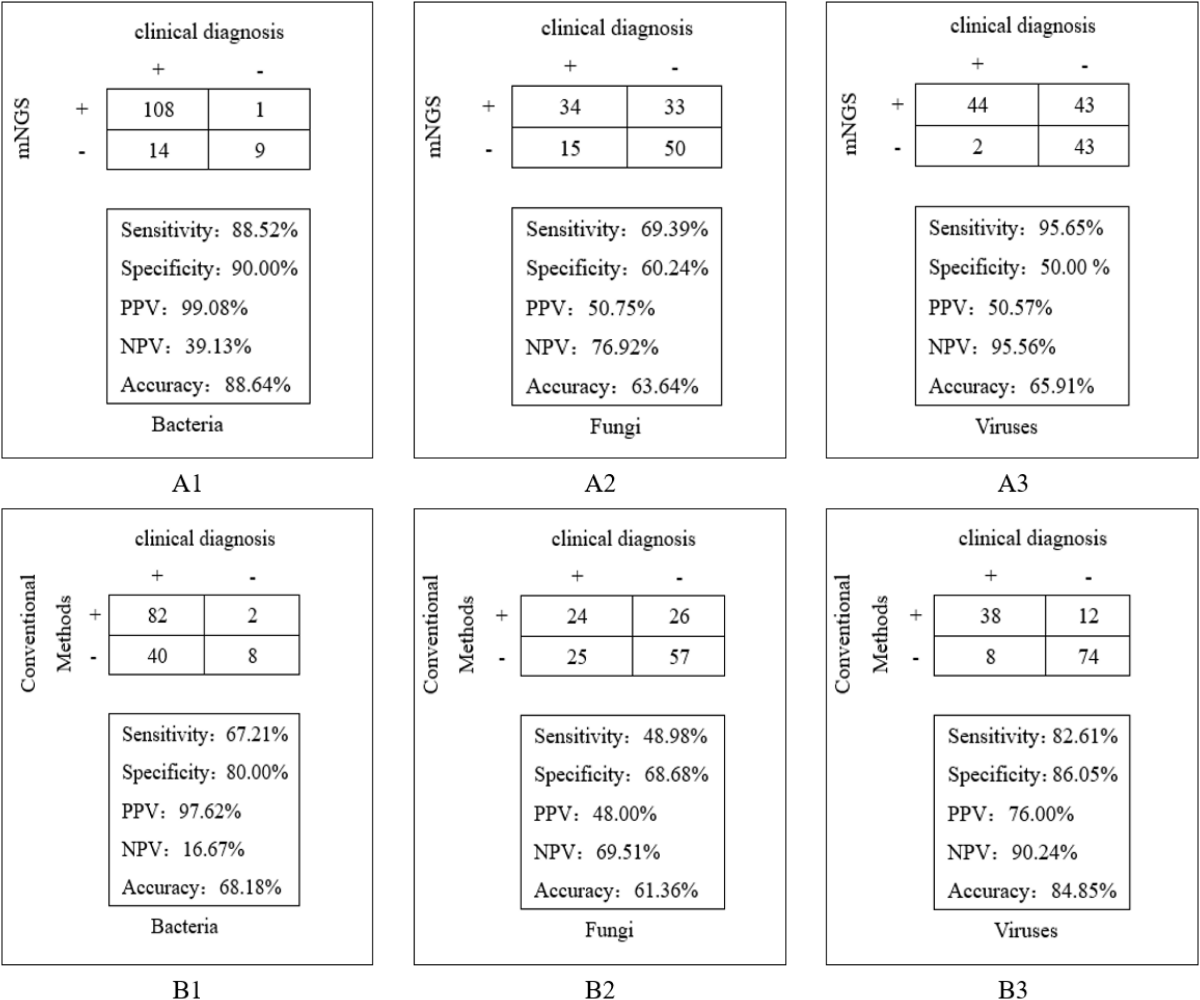
Comparison of clinical diagnostic effects between mNGS and conventional methods. **(A1)** The detection efficiency of mNGS in bacteria; **(A2)** The detection efficiency of mNGS in fungi; **(A3)** The detection efficiency of mNGS in viruses. **(B1)** The detection efficiency of conventional methods in bacteria; **(B2)** The detection efficiency of conventional methods in fungi; **(B3)** The detection efficiency of conventional methods in viruses.

The detection efficiency of mNGS and conventional methods in clinical diagnosis was compared. The results demonstrated that mNGS exhibited superior sensitivity across pathogen types: 88.52% vs. 67.21% for bacteria (χ^2^ = 17.361, *P*=0.000), 69.39% vs. 48.98% for fungi, and 95.65% vs. 82.61% for viruses, with statistically significant differences observed specifically in bacterial detection. Moreover, mNGS showed higher accuracy in bacteria and fungi: 88.64% vs. 68.18% for bacteria (χ^2^ = 17.333, *P*=0.000), 63.64% vs. 61.36% for fungi, with statistically significant differences in bacterial detection (**Figure 4**).

## 4 Discussion

The clinical manifestations of SP are varied and the pathogens are complex in different groups. Community-acquired pneumonia is mainly caused by *H. influenzae*, *S. pneumoniae* and *K. pneumoniae*, and approximately 27%-39% is viral pneumonia, especially in the context of the current influenza and 2019-nCov epidemics (Zhan et al., 2014; Qu et al., 2015; Zhou et al., 2019). The pathogens of SP patients with diabetes are mainly *K. pneumoniae*, *E. coli*, *P. aeruginosa* and *S. aureus* (Wang et al., 2020). In patients with SP who are at risk of aspiration, the main pathogens are *K. pneumoniae, P. aeruginosa* and *A. baumannii* (Patil et al., 2021). In the 1970s, anaerobic bacteria were also the main pathogens of aspiration pneumonia, but recent studies have shown that the detection rate of anaerobic bacteria has decreased (Marik, 2001). Special populations such as immunocompromised hosts or long-term hormone application are more susceptible to specific pathogenic microbial infections such as fungi and tuberculosis (He, 2008; Liu et al., 2011; Wahab et al., 2024).

In our study, the median age of patients was 70 years old, and most of the patients were comorbid with chronic underlying diseases such as diabetes mellitus, cerebral infarction, or the presence of dysphagia or bedridden conditions. Both conventional methods and mNGS in bacterial detection suggested that *K. pneumoniae*, *A. baumannii*, and *P. aeruginosa* were the main causative organisms, which was in line with previous relevant studies (Patil et al., 2021). However, mNGS is superior to conventional methods for *S. aureus*, *S. pneumoniae*, *H. influenzae* and *E. coli*. In addition, our research also showed that mNGS is easier to detect pathogens such as *MTBC*, *NTM*, *L. pneumophila*, *C. psittaci*, and anaerobic bacteria than traditional methods, and which may provide a reference for early targeted anti-infection therapy in the clinic. In terms of fungi, both the conventional method and mNGS detected a higher number of Candida, whereas normally, it obtained from airway secretions may only indicate colonization. It is worth noting that mNGS has high sensitivity for fungi that are difficult to detect through traditional methods, including Aspergillus, Pneumocystis jirovecii and Rhizomucor pusillus, which can provide an effective reference for clinical antifungal treatment. In terms of viruses, conventional methods detected more influenza viruses than mNGS, which may be related to the differences in specimen collection sites and detection methods. A positive test result obtained by traditional methods of detecting antigens or antibodies through nasopharyngeal swabs or serum indicates the presence of upper respiratory tract infection or a recent viral infection history, but cannot confirm lower respiratory tract viral infection. In contrast, although the influenza virus detection rate of mNGS is lower than that of conventional methods, its positive results clearly indicate that it is the causative agent of lower respiratory tract infection. In addition, our study also found that compared with traditional methods, mNGS is more likely to indicate mixed infections. Of the 37 COVID-19 positive cases 14 (37.84%) had concurrent detection of *A. baumannii*, 11 (29.73%) had concurrent detection of *K. pneumoniae*, and 13 (35.14%) had concurrent detection of Aspergillus. Of the 10 influenza virus-positive cases, *P. aeruginosa* was detected concurrently in 6 and Aspergillus in 4. Nine out of 44 K*. pneumoniae* infections were positive for Aspergillus, 10 out of 35 P*. aeruginosa* infections were positive for Aspergillus, and 6 out of 16 Enterococci -positive cases were positive for Aspergillus. This may indicate that a larger proportion of adult patients with SP are infected by a mixture of pathogenicmicroorganisms.

The mNGS has many advantages such as wide detection range, high sensitivity, and short detection time. Chen Hongbin et al. reported that the pathogen spectrum detected by BALF mNGS was wider than that of culture, more fungi and viruses were detected, and the detection time of mNGS (2 days) was shorter than that of culture (bacteria 3 days, fungi 7 days, mycobacteria 45 days) (Chen et al., 2020). But it also has certain limitations, such as molecular biology cannot be used as a standard diagnosis. The respiratory tract is colonized by a large number of microorganisms. Detecting microorganisms in respiratory samples through mNGS requires identifying suspected pathogenic microorganisms, colonizing microorganisms, or contaminating microorganisms (Langelier et al., 2018). Clinicians need to determine the pathogen based on patient’s clinical manifestations, immune status, underlying diseases, etc. In our study, mNGS detected high levels of *S. maltophilia*, *C. striatum*, Enterococcus and Candida, but they are usually conditional pathogens and rarely cause severe lower respiratory tract infections. Human herpesviruses and Torque teno virus have been detected in many cases, but usually they do not cause pneumonia and have limited value in guiding clinical treatment. In addition, it is far from satisfactory to provide the pathogen identification results alone. It is necessary to detect clinically relevant antibiotic resistance genes (ARGs) and further predict pathogen resistance to guide patient management. Although culture-independent mNGS is attractive, but most mNGS diagnosis platforms are based on short reads sequencing, it is challenging to determine the detected ARGs originated from the genome of the causative agent rather than normal flora, or contaminations in environment (Langelier et al., 2018). There are still many obstacles to overcome.

Early identification of pathogens and targeted anti-infective measures are important tools to reduce mortality and improve prognosis in patients with severe pneumonia. Metagenomic next-generation sequencing has been increasingly used in the diagnosis of infectious diseases, particularly when conventional diagnostic approaches have limitation. Compared with conventional methods, mNGS has unique advantages in detecting pathogenic microorganisms such as Mycobacterium, *L. pneumophila*, anaerobic bacteria, fungi and viruses, and is becoming one of the important microbiological testing tools in the clinic. Nevertheless, more application practice is still needed for different patients, samples, and scenarios, and it needs to be combined with traditional testing methods for more rational interpretation.

## Data Availability

The raw data supporting the conclusions of this article will be made available by the authors, without undue reservation.
